# Xpert MTB/XDR implementation in South Africa: cost outcomes of centralised vs. decentralised approaches

**DOI:** 10.5588/ijtldopen.23.0501

**Published:** 2024-05-01

**Authors:** N. Cassim, S.V. Omar, S.D. Masuku, H. Moultrie, W.S. Stevens, F. Ismail, P. da Silva

**Affiliations:** ^1^ National Priority Programme, National Health Laboratory Service, Johannesburg,; ^2^ Wits Diagnostic Innovation Hub, Faculty of Health Sciences, University of Witwatersrand, Johannesburg,; ^3^ Centre for Tuberculosis, National TB Reference Laboratory, National Institute for Communicable Diseases, Division of the National Health Laboratory Service, Johannesburg,; ^4^ Department of Medical Microbiology, University of Pretoria, Pretoria,; ^5^ Health Economics and Epidemiology Research Office, Faculty of Health Sciences, University of the Witwatersrand, Johannesburg, South Africa

**Keywords:** drug susceptibility testing, rifampicin-resistant tuberculosis, multidrug-resistant tuberculosis, line-probe assay

## Abstract

**INTRODUCTION:**

In South Africa, Xpert^®^ MTB/RIF Ultra (Ultra) is the recommended diagnostic assay for TB with line-probe assays for first- (LPAfl) and second-line drugs (LPAsl) providing additional drug susceptibility testing (DST) for samples that were rifampicin-resistant (RR-TB). To guide implementation of the recently launched Xpert^®^ MTB/XDR (MTB/XDR) assay, a cost-outcomes analysis was conducted comparing total costs for genotypic DST (gDST) for persons diagnosed with RR-TB considering three strategies: replacing LPAfl/LPAsl (centralised level) with MTB/XDR vs. Ultra reflex testing (decentralised level). Further, DST was performed using residual specimen following RR-TB diagnosis.

**METHODS:**

The total cost of gDST was determined for three strategies, considering loss to follow-up (LTFU), unsuccessful test rates, and specimen volume.

**RESULTS:**

For 2019, 9,415 persons were diagnosed with RR-TB. A 35% LTFU rate between RR-TB diagnosis and LPAfl/LPAsl-DST was estimated. Unsuccessful test rates of 37% and 23.3% were reported for LPAfl and LPAsl, respectively. The estimated total costs were $191,472 for the conventional strategy, $122,352 for the centralised strategy, and $126,838 for the decentralised strategy. However, it was found that sufficient residual volume for reflex MTB/XDR testing is a limiting factor at the decentralised level.

**CONCLUSION:**

Centralising the implementation of XDR testing, as compared to LPAfl/LPAsl, leads to significant cost savings.

## RÉSUMÉ

**INTRODUCTION:**

En Afrique du Sud, Xpert^®^ MTB/RIF Ultra (Ultra) est le test de diagnostic recommandé pour la TB avec des tests par sonde de ligne pour les médicaments de première (LPAfl) et de deuxième ligne (LPAsl) fournissant des tests de sensibilité aux médicaments (DST) supplémentaires pour les échantillons résistants à la rifampicine (RR-TB). Afin d'orienter la mise en œuvre du test Xpert^®^ MTB/XDR (MTB/XDR) récemment lancé, une analyse coûts-résultats a été réalisée en comparant les coûts totaux de la DST génotypique (gDST) pour les personnes diagnostiquées avec une RR-TB en tenant compte de trois stratégies : remplacer le LPAfl/LPAsl (niveau centralisé) par le MTB/XDR par rapport au test Ultra reflex (niveau décentralisé). De plus, l'heure d'été a été réalisée à l'aide d'un échantillon résiduel après le diagnostic de RR-TB.

**MÉTHODES:**

Le coût total de la gDST a été déterminé pour trois stratégies, en tenant compte de la perte de suivi (LTFU), des taux d'échec des tests et du volume d'échantillons.

**RÉSULTATS:**

En 2019, 9 415 personnes ont reçu un diagnostic de RR-TB. Un taux de LTFU de 35% entre le diagnostic de RR-TB et le diagnostic de LPAfl/LPAsl-DST a été estimé. Des taux d'échec de 37% et de 23,3% ont été signalés pour LPAfl et LPAsl, respectivement. Les coûts totaux estimés étaient de 191 472 dollars pour la stratégie conventionnelle, de 122 352 dollars pour la stratégie centralisée et de 126 838 dollars pour la stratégie décentralisée. Cependant, il a été constaté qu'un volume résiduel suffisant pour les tests réflexes MTB/XDR est un facteur limitant au niveau décentralisé.

**CONCLUSION:**

La centralisation de la mise en œuvre des tests XDR, par rapport à LPAfl/LPAsl, permet de réaliser d'importantes économies.

Globally, TB is a major cause of ill health and mortality.^1^ There were respectively 7.1, 5.8 and 6.4 million people newly diagnosed with TB in 2019, 2020 and 2021.^2–4^ In South Africa, the incidence of TB was 513/100,000 population in 2021.^4,5^ The prevalence of bacteriologically confirmed pulmonary TB was 852/100,000 population (≥15 years) in 2018.^6^ Furthermore, HIV co-infection rate among TB cases was 59% compared to 8% globally.^1,3,6^

A local survey conducted between 2012 and 2014 reported that the prevalence of multidrug-resistant TB (MDR-TB) was 2.1% for new and 4.6% for retreatment cases.^7,8^ The prevalence of extensively drug-resistant TB (XDR-TB) among individuals with MDR-TB, using the definition of resistance to both a fluoroquinolone (FQ) and second-line injectable drug (SLID), was 4.9%.^3,8,9^ While the rate of MDR-TB has remained stable from earlier surveys, the prevalence of rifampicin-resistant TB (RR-TB) has increased from 3.4% (2001–2002) to 4.6% (2012–2014).^7,8^

The 2019 local guidelines recommend the diagnosis of rifampicin-resistant (RR-TB) through the use of the Xpert^®^ MTB/RIF Ultra (Ultra; Cepheid, Sunnyvale, CA, USA).^10^ For those with confirmed RR-TB, additional testing is performed on a second specimen as part of the drug resistance TB reflex workflow at the centralised level, which included digestion and decontamination as per standard protocol,^11,12^ with the following tests performed on sediment: 1) smear microscopy, 2) GenoType MTBDR*plus* v2.0 (Hain Lifescience, Nehren, Germany) for first-line genotypic drug susceptibility testing (gDST), that is, first-line line-probe assay (LPAfl) (Bruker; Billerica, MA, USA), 3) GenoType MTBDR*sl* v2.0 (Hain Lifescience) for second-line gDST (or second-line LPA [LPAsl]) (Bruker), 4) TB culture, and 5) culture-based phenotypic drug susceptibility testing (pDST), i.e., conventional workflow.

In July 2020, the Xpert^®^ MTB/XDR (MTB/XDR; Cepheid) test enabled the expanded gDST profiling in under 90 min.^13^ This assay is intended as a reflex test to detect genotypic resistance to isoniazid (INH), FQ, ethionamide (ETH) and SLIDs following a positive RR-TB Ultra test.^13^ A study that compared MTB/XDR concordance with pDST reported sensitivity and specificity >90% for INH, FQ, amikacin (AMK) and kanamycin (KM).^13^ For capreomycin (CPM) and ETH, a sensitivity of ≤70% was reported with specificities ≥97.3%.^13^ An analysis of MTB/XDR against pDST reported specificities of 94% (INH), 95% (FQ), 54% (ETH), 73% (AMK), 86% (KM), and 61% (CPM).^14^ A manufacturer-independent validation study confirmed MTB/XDR to be a reliable and sensitive assay for expanded resistance detection.^15^ Further, MTB/XDR has been shown to have higher sensitivity in smear-negative sputum specimens compared to LPAfl/LPAsl.^13,14^

In consideration for the introduction of the MTB/XDR assay to South Africa’s existing GeneXpert footprint, the objective of this study was to conduct a cost-outcomes analysis that estimated the cost/person and cost/successful gDST result in those diagnosed with RR-TB under two strategies: 1) deployment of the MTB/XDR assay as a replacement for LPAfl and LPAsl at centralised laboratories, herein referred to as ‘centralised strategy’, and 2) deployment of the assay as a reflex test from Ultra, at decentralised level, herein referred to as ‘decentralised strategy’. In addition, the same outcomes were estimated for the conventional workflow. All three strategies apply when a RR-TB diagnosis is made using Ultra.

## METHODS

The Consolidated Health Economic Evaluation Reporting Standards checklist was used in the preparation of the manuscript.^16,17^ The study population were persons with laboratory confirmed RR-TB.

Sequence of events within each workflow and implementation strategy:

1) Conventional workflow: second specimen referred to centralised TB culture laboratories for digestion and decontamination with TB culture incubated in parallel and LPAfl performed on sediment. LPAsl testing follows if LPAfl is successful. Where LPAfl is unsuccessful, both LPAfl and LPAsl are repeated off cultured isolate. Where LPAfl (tested on sediment) is successful but LPAsl testing is unsuccessful, LPAsl is repeated off cultured isolate.
2) Centralised strategy: referral of the second specimen to the network of centralised laboratories in the conventional workflow and similar specimen processing, except that MTB/XDR replaces LPAfl and LPAsl testing. Where MTB/XDR is unsuccessful, testing is repeated from cultured isolate.
3) Decentralised strategy: this option considers decentralisation across >160 Xpert testing laboratories as a reflex test from residual specimen. Where residual specimen is insufficient, MTB/XDR is tested on a second specimen: either retained by the laboratory (where two-baseline specimen collection strategy is active) or requires additional specimen collection.

### Model design, data sources and parameters

**Figure 1.**
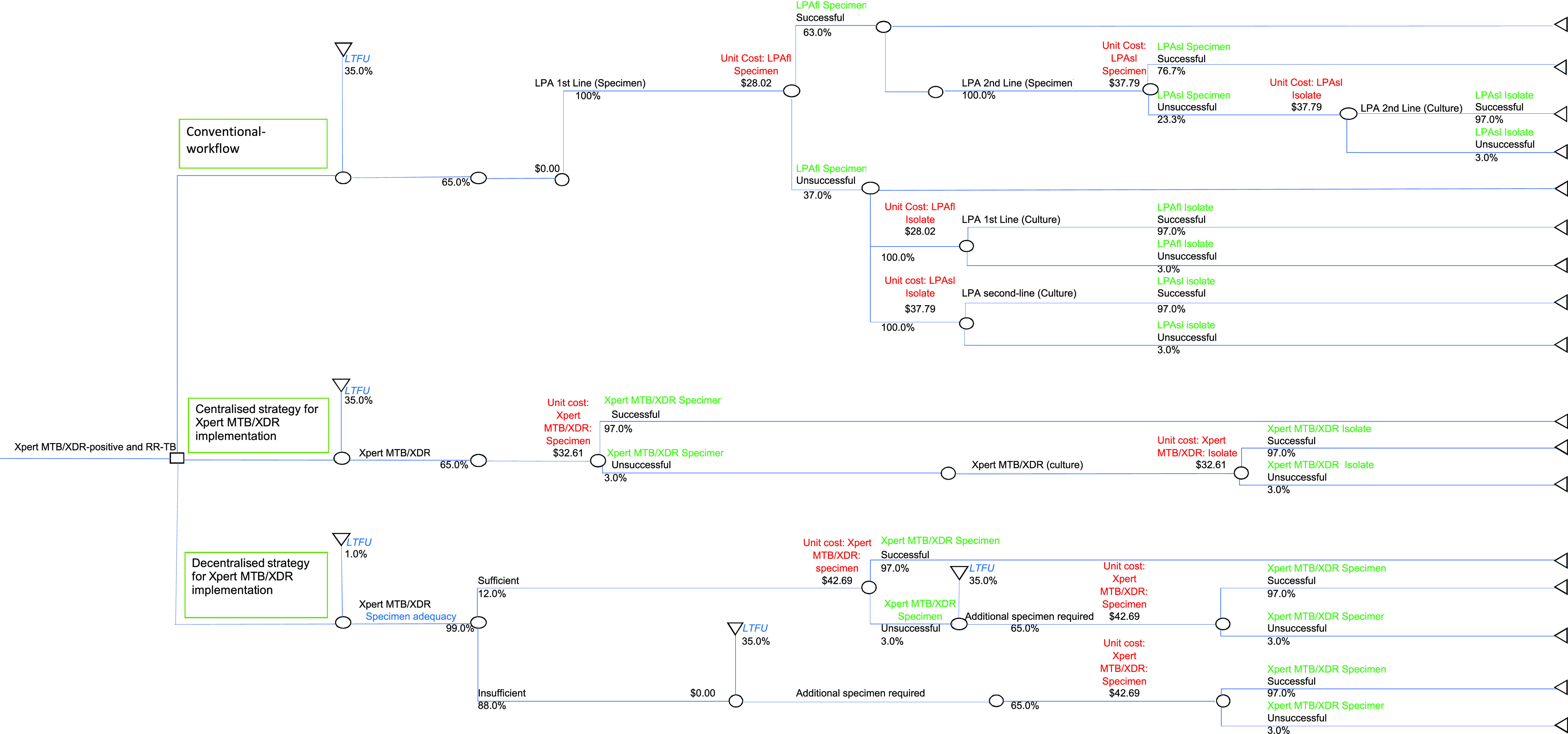
Decision tree for the cost outcomes analysis for the three diagnostic arms: 1) conventional workflow; 2) the centralised strategy for the implementation of MTB/XDR testing, and 3) the decentralised strategy for the implementation of MTB/XDR testing. For each diagnostic arm, the LTFU and success rates are used based on data described in Table 1. Captions have been provided to indicate what each value represents. Note that numbers in red represent unit costs, numbers in green show successful/unsuccessful rates, and blue indicates LTFU and specimen adequacy. LTFU = loss to follow-up; LPAfl = GenoType MTBDR*plus* v2.0 for first-line gDST; LPAsl = GenoType MTBDR*sl* v2.0 for second-line gDST; gDST = genotypic drug susceptibility testing.

We constructed a decision tree using MS Excel (Microsoft; Redmond, WA, USA), which analysed the costs and outcomes of the conventional workflow, as well as two implementation strategies. This analysis was based on 2019 data. The decision tree incorporated variables such as specimen adequacy, test unsuccessful rates, losses at various stages of the diagnostic cascade and unit costs (Figure 1).

For each workflow, the following were estimated: 1) the average cost/person; 2) average cost/successful result; and 3) the total cost of gDST given the losses at various stages, unsuccessful test rates and specimen adequacy parameters. Individuals transitioned from one diagnostic state to the next, i.e., RR-TB diagnosis, followed by gDST, across all workflows. The model did not follow-up persons for any specific time interval. In addition, the number of individuals diagnosed with RR-TB that would receive successful gDST results was estimated. The decision tree was used to determine the rolled-back probabilities, rolled-back costs and total costs.

Several data sources were utilised: 1) Electronic Drug-Resistant Tuberculosis Register (EDRWeb) and the National Health Laboratory Service’s (NHLS) Corporate Data Warehouse (CDW). EDRWeb is the national electronic treatment register for those initiated on a drug-resistant TB (DR-TB) regimen containing individual-level data from facility TB registers.^17–20^ EDRWeb provided estimates of RR-TB cases,^18–21^ number of individuals initiated on DR-TB, and probabilities of loss to follow-up (LTFU). The latter probability was applied to the conventional workflow and centralised strategy. However, as the decentralised strategy is based on MTB/XDR testing from residual specimens on Ultra-diagnosed RR-TB, a lower LTFU of 1% was assumed (based on reflex cryptococcal antigen testing).^22^

Unit costs were sourced from an ingredients-based (reagents and test consumables) costing analysis undertaken for testing previously performed at a high-throughput centralised laboratory within the Gauteng Province. Costs were obtained from the supply chain management and manufacturer-supplied quotations. All costs were determined in South African rands and reported in United States dollars (USD) using the average annual exchange rate of 14.03.^23^ A provider perspective was taken with all costs reported as the testing provider.

To calculate the staffing annual equivalent cost (AEC), the cost/minute, total hands-on time and annual test volumes were used. The number and category of staff that performed testing was used with NHLS mid-point cost-to-company (CTC) salary scales to determine the cost/minute.

For laboratory equipment, working life years were set at 5 years with a discount rate of 4% for the AEC calculation. Annual placement, service and callout costs were also included (where applicable). Where laboratory equipment was shared, a percentage allocation was applied.

For gDST, the reagents consisted of the MTBDR*plus* and MTBDR*sl* kits. The MTB/XDR reagent cost was provided by Cepheid. Cost for the respective test consumables were obtained from the NHLS ERP. The cost of MGIT™ (Mycobacteria Growth Indicator Tube; BD, Franklin Lakes, NJ, USA) culture was excluded as local guidelines indicate that specimens from patients diagnosed with RR-TB will be sent for culture, irrespective of the gDST results.^10^ Therefore, we excluded this economic cost as it would be incurred by the healthcare system due to the sequence of testing performed when ‘DR-TB: Reflex DST Testing’ is indicated on the request form.^10^ The cost of specimen collection materials was also assessed. For reagents, test consumables and specimen collection materials, an error rate of 3% and 6% was applied for the MTB/XDR and gDST, respectively. Overhead costs were excluded from the analysis as they were out of the scope of the study.

The unit costs were determined by dividing the AEC by annual tested volumes. This analysis was based on test volumes for the Braamfontein Laboratory, Braamfontein, South Africa. For the conventional workflow, test volumes were determined from specimen-level data and included referrals from surrounding laboratories to the centralised laboratory. For the centralised strategy, gDST volumes for Gauteng Province were used (referrals from 14 peripheral laboratories to the centralised laboratory). For the decentralised strategy, it was assumed that testing would only be offered for surrounding health facilities and excluded inter-laboratory referrals.

To determine the robustness of the model, a one-way sensitivity analysis of key parameters was conducted to assess the impact on the average cost/successful gDST result. The parameters varied included LTFU and unit costs.

Ethical approval was granted by the Human Research Ethics Committee (Medical) at the University of Witwatersrand, Johannesburg, South Africa (M160978), patient consent was not required.

## RESULTS

There were 9,415 individuals reported on EDRWeb who were initiated on a DR-TB treatment regimen. The LTFU for an RR-TB diagnosis and gDST was estimated to be 35% (Table 1).^20^ Therefore, for all steps in the decision tree where a second-sputum specimen was requested, the LTFU was assumed to be 35% for all strategies (Figure 1). The analysis of national-level specimen data revealed rates of unsuccessful tests of respectively 37% and 23.3% for LPAfl and LPAsl on a second-sputum specimen. The proportion of unsuccessful LPAsl tests was lower than that of LPAfl tests, as LPAsl was not conducted on specimens where LPAfl testing had not yielded a successful result. For gDST on cultured isolate, unsuccessful test rates of 3% were reported compared to 3.0% for MTB/XDR (Table 1). The specimen adequacy survey reported that only 12% met the stipulated criteria in the decentralised strategy. The unit costs were based on annual tested volumes of 2,116 and 1,009 for LPAfl and LPAsl, respectively, with measurements in USD. The annual tested volumes estimated for the centralised and decentralised strategy were respectively 1,290 and 193.

**Table 1.** Decision model input parameters used to determine the transition probabilities between diagnostic states.*

Parameters	Value used	Source
Number of persons with rifampicin-resistant TB	9,415	EDRWeb
LTFU: gDST and MTB/XDR test	35.0%	EDRWeb
MTBDR*plus* (LPAfl) unsuccessful rates (tested on specimen)	37.0%	Analysis of specimen-level data (CDW)
MTBDR*sl* (LPAsl) unsuccessful rates (tested on specimen)	23.3%	Analysis of specimen-level data (CDW)
MTBDR*plus* (LPAfl) unsuccessful rates (tested on cultured isolate)	3.0%	Analysis of specimen-level data (CDW)
MTBDR*sl* (LPAsl) unsuccessful rates (tested on cultured isolate)	3.0%	Analysis of specimen-level data (CDW)
LTFU: reflexing to MTB/XDR from Ultra (decentralised strategy)	1.0%	Analysis of cryptococcal antigen reflex testing (CDW)
Specimen adequacy: reflexing to MTB/XDR from Ultra (decentralised strategy)	12.0%	Survey of samples from 14 Xpert laboratories
MTB/XDR unsuccessful rates	2.96%	Penn-Nicholson et al. and Omar et al.
MTB/XDR unsuccessful rates (tested on cultured isolate)	3.0%	Analysis of specimen-level data (CDW)

* The decision tree was used to assess diagnostic states for the three study arms, as follows: 1) conventional workflow or comparator; 2) the centralised strategy for MTB/XDR, implementation; and 3) the decentralised strategy for MTB/XDR implementation. The parameters were applied to LPAfl, LPAsl and MTB/XDR.

EDRWeb = Electronic Drug-Resistant Tuberculosis Register; LTFU = loss to follow-up; gDST = genotypic drug susceptibility testing; LPAfl = GenoType MTBDR*plus* v2.0 for first-line gDST; CDW = corporate data warehouse; LPAsl = GenoType MTBDR*sl* v2.0 for second-line gDST.

The ingredients-based costing analysis estimated unit costs of respectively USD28.02 and USD37.79 for LPAfl and LPAsl testing (Table 2) for direct specimen testing. The total unit cost for gDST (tested on specimen) was USD65.80. The same unit costs were used for gDST tested on culture isolates. The centralised and decentralised strategies reported a unit cost of respectively USD32.61 and USD42.69.

**Table 2.** Unit costs for gDST tests.

Test	Unit	Unit cost (USD 2019)
MTBDR*plus* (LPAfl), tested on specimen	1 test	28.02
MTBDR*plus* (LPAfl), tested on culture isolate	1 test	28.02
MTBDR*sl* (LPAsl), tested on specimen	1 test	37.79
MTBDR*sl* (LPAsl), tested on culture isolate	1 test	37.79
Centralised strategy: MTB/XDR	1 test	32.61
Decentralised strategy: MTB/XDR	1 test	42.69

gDST = genotypic drug susceptibility testing; USD = US dollar; LPAfl = GenoType MTBDR*plus* v2.0 for first-line gDST; LPAsl = GenoType MTBDR*sl* v2.0 for second-line gDST.

The cost-outcome analysis reported a total cost for gDST of respectively USD191,472, USD122,352 and USD126,838 for the conventional workflow, and the centralised and decentralised strategies (Table 3). Of those diagnosed with RR-TB, 64.0% received a successful gDST result for the conventional workflow. The model estimates that respectively 64.3% and 66.7% of patients with RR-TB would receive successful gDST in the centralised and decentralised strategies.

**Table 3.** Number of persons with RR-TB who received a successful gDST (LPAfl and LPAsl) for three arms, as follows: 1) conventional workflow; 2) centralised strategy for MTB/XDR implementation; and 3) decentralised strategy for MTB/XDR implementation. The total costs for each arm, the average cost per person and per successful gDST result, are reported.

Diagnostic algorithm	Persons with RR-TB *n*	Persons with a successful gDST result *n* (%)	Total costs (USD)	Average cost/person (USD)	Average cost/successful gDST result (USD)
Conventional workflow	9,415	6,025 (64.0)	191,472	78.24	70.15
Centralised strategy MTB/XDR*	9,415	6,114 (64.9)	122,352	21.82	21.78
Decentralised strategy MTB/XDR^†^	9,415	6,280 (66.7)	126,838	29.29	28.57

* Includes referrals from surrounding laboratories.

† Offered immediately after Ultra testing to only surrounding health facilities.

RR-TB = rifampicin-resistant TB; gDST = genotypic drug susceptibility testing; LPAfl = GenoType MTBDR*plus* v2.0 for first-line gDST; LPAsl = GenoType MTBDR*sl* v2.0 for second-line gDST; USD = US dollar.

The average cost/person was respectively USD78.24, USD21.82 and USD29.29 for the conventional workflow, and the centralised and decentralised strategies (Table 3). In comparison, the average cost/successful gDST result was respectively USD70.15, USD21.78 and USD28.57.

**Figure 2.**
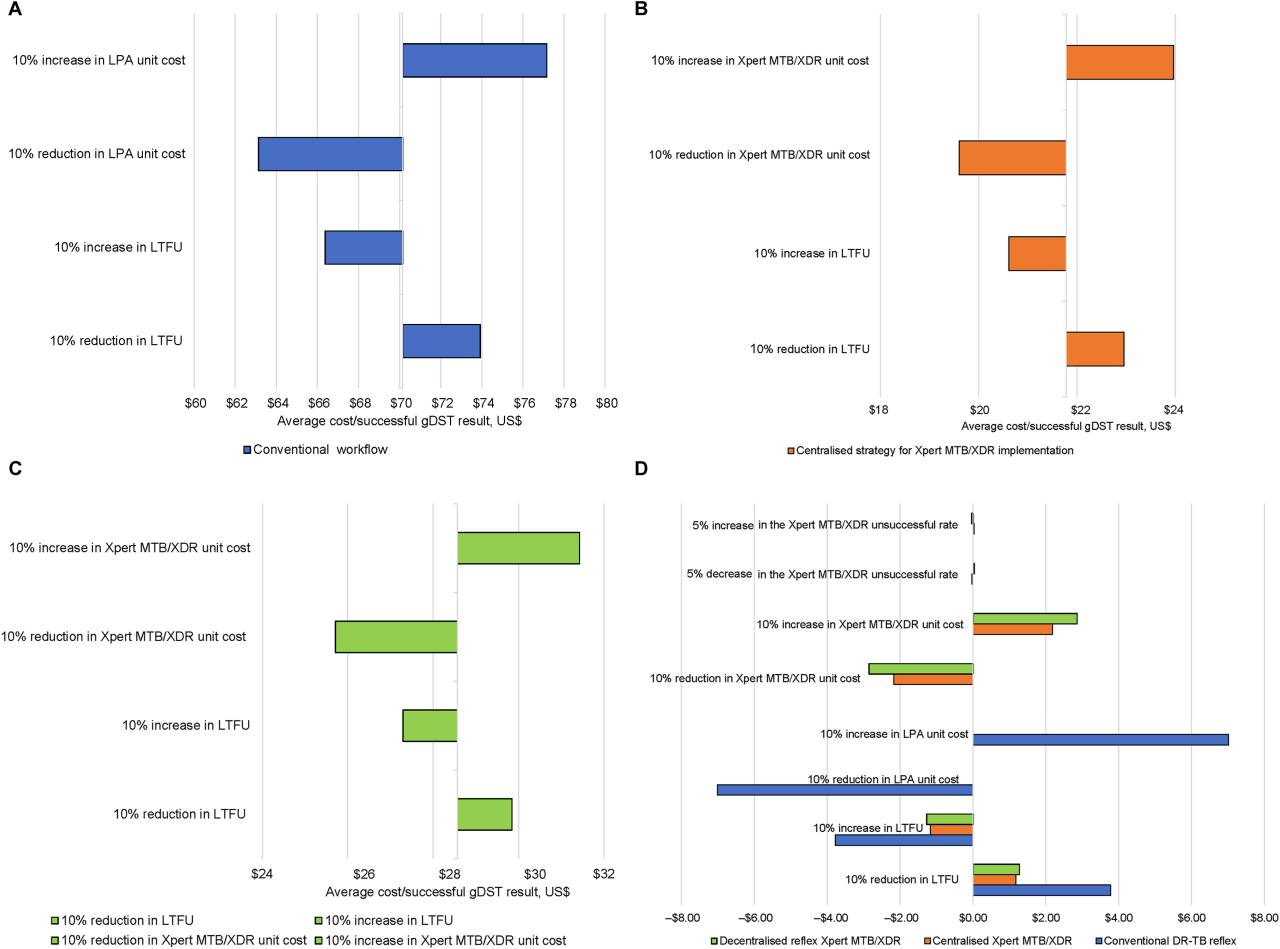
One-way sensitivity to assess the impact on the average cost/successful gDST results by varying unit costs and LTFU by 10% for three diagnostic arms: 1) conventional workflow; 2) centralised strategy for the implementation of MTB/XDR testing; and 3) decentralised strategy for the implementation of MTB/XDR testing. The difference for the reported average cost per successful result for each scenario was compared to the results of the one-way sensitivity. LPA = line-probe assay; LTFU = loss to follow-up; DR-TB = drug-resistant TB.

For the conventional workflow, a 10% reduction in unit costs resulted in a saving of USD7.02 compared to USD3.78 for a 10% improvement in LTFU (Figure 2A). A 10% improvement in unit costs and LTFU resulted in a cost saving of USD2.18 and USD1.17, respectively, for the centralised strategy (Figure 2B). For the decentralised strategy, a saving of respectively USD2.86 and USD1.27 was reported for a 10% improvement in unit costs and LTFU (Figure 2C). Sensitivity analysis demonstrated that the average cost/successful gDST for all three workflows was most sensitive to changes in unit costs (Figure 2D).

## DISCUSSION

The objective of this study was to conduct a cost-outcomes analysis comparing the total cost for gDST testing for those diagnosed with RR-TB for two options: 1) centralised strategy and 2) decentralised strategy, compared to the conventional workflow.

A unit cost of USD28.02 and USD37.79 was estimated for LPA*fl* and LPA*sl,* respectively (total unit cost of USD65.80)*.* The centralised strategy reported a unit cost of USD32.61 compared to USD42.69 for the decentralised strategy. For the conventional workflow, a total cost of USD191,472 was estimated. In contrast, the centralised and decentralised strategy reported total costs of respectively USD122,352 and USD126,838. These findings indicate that at the national level, replacing LPAfl/LPAsl with MTB/XDR could lead to annual cost savings of up to USD69,120 based on the centralised strategy. The average cost/successful gDST result was USD48.37 higher for the conventional workflow when compared to the centralised strategy, further highlighting substantial cost savings by the replacement approach. Bainomugisa et al. reported that MTB/XDR results are available rapidly as opposed to several weeks with traditional culture-based methods or several days with LPA assays.^24^ A substantial reduction in turnaround time (TAT) would reduce the time to MDR- and pre-extensively drug-resistant TB diagnosis and treatment initiation.

The one-way sensitivity analysis revealed that changes to LTFU or test unit costs would only affect the average cost/successful gDST result by less than USD7.00. This indicates that to effect substantial changes to the average cost, differences in excess of 20% are required for these parameters. With regard to LTFU, >30% of those diagnosed with RR-TB did not receive gDST results based either on an unsuccessful result or failure to submit a second-sputum specimen for testing. Therefore, potential MDR- or pre-XDR-TB diagnoses may be missed and changes to treatment regimens not implemented. The decentralised strategy has the potential to address this gap. Unfortunately, the low specimen adequacy rates would necessitate collection of a second specimen for MTB/XDR testing as opposed to the test being conducted as a reflex on residual specimen following Ultra testing. Reflex testing is ideal, and local data indicate its feasibility in that >98% of CD4 specimens (<100 cells/µl) receive a reflex cryptococcal antigenaemia (CrAg) testing using remnant blood. Reflex CrAg testing has shown similar reductions in cryptococcal meningitis to health facility point-of-care testing.^25^ However, sputum collection is challenging, and due to the impact of specimen adequacy (particularly meeting the minimum required volume) and the high rates thereof encountered, insufficient residual specimen would be available for reflex MTB/XDR testing, impacting the decentralised offering. One contributing factor is the upfront single-sputum collection strategy implemented in eight out of nine provinces in South Africa. Adopting an upfront two-sputum collection strategy could address the issue of insufficient residual volume for reflex testing with the MTB/XDR assay. Under this programmatic change, the decentralised strategy may be favoured as the MTB/XDR results would follow shortly after diagnosis of RR-TB.

Regarding unit costs, further reductions would only be possible through supplier negotiation. Our findings reveal that the centralised strategy results in lower costs than the decentralised strategy due to higher test volumes. In a developing context, a saving of USD6.79 in the average cost/successful gDST result between the ‘centralised’ and ‘decentralised’ approaches is substantial. In conclusion, our findings reveal that centralised switching to MTB/XDR for second-line gDST would result in public health expenditure cost savings with the potential to improve health outcomes due to improved test success rates and TAT for earlier clinical management.

This study did not assess the impact on subsequent TB treatment costs. As there was a small increase in the number of persons with a successful gDST result for the MTB/XDR strategy, it is anticipated that slightly higher treatment costs would be incurred. This is due to differences in LTFU, unsuccessful rates and specimen adequacy would also have consequences for treatment costs.

### Limitations

Overhead costs were excluded as they are calculated for the entire organisation and difficult to assign for one test. It is anticipated that for the decentralised strategy higher overhead costs may be reported due to lower test volumes. A high LTFU was reported between RR-TB diagnosis and DST on a second specimen. The costs associated with these patients returning for care were not factored in. This study did not evaluate costs related to introduction of the new algorithms.
